# A Distributed Control Scheme Using SiC-Based Low Voltage Ride-Through Compensator for Wind Turbine Generators

**DOI:** 10.3390/mi13010039

**Published:** 2021-12-28

**Authors:** Chao-Tsung Ma, Zong-Hann Shi

**Affiliations:** Applied Power Electronics Systems Research Group, Department of EE, CEECS, National United University, Miaoli City 36063, Taiwan; M0921008@o365.nuu.edu.tw

**Keywords:** wind turbine generator (WTG), permanent magnet synchronous generator (PMSG), low voltage ride-through (LVRT), silicon carbide (SiC)-based inverter

## Abstract

As the penetration of renewable energy power generation, such as wind power generation, increases low voltage ride-through (LVRT), control is necessary during grid faults to support wind turbine generators (WTGs) in compensating reactive current to restore nominal grid voltages, and maintain a desired system stability. In contrast to the commonly used centralized LVRT controller, this study proposes a distributed control scheme using a LVRT compensator (LVRTC) capable of simultaneously performing reactive current compensation for doubly-fed induction generator (DFIG)-, or permanent magnet synchronous generator (PMSG)-based WTGs. The proposed LVRTC using silicon carbide (SiC)-based inverters can achieve better system efficiency, and increase system reliability. The proposed LVRTC adopts a digital control scheme and dq-axis current decoupling algorithm to realize simultaneous active/reactive power control features. Theoretical analysis, derivation of mathematical models, and design of the control scheme are initially conducted, and simulation is then performed in a computer software environment to validate the feasibility of the system. Finally, a 2 kVA small-scale hardware system with TI’s digital signal processor (DSP) as the control core is implemented for experimental verification. Results from simulation and implementation are in close agreement, and validate the feasibility and effectiveness of the proposed control scheme.

## 1. Introduction

In recent years, the worldwide demand for energy has been rapidly growing to support the development of various modern technologies; thus, the vast use of fossil fuels has negatively affected the environment. Therefore, renewable energy (RE)-based distributed power generation (DG) has been intensively researched in the past decade. In the near future, the penetration of various grid-connected DG systems is expected to keep increasing. As a result, utilizing advanced power converter-based compensators to work with RE-based generation systems and existing grids are one of the most important tasks in ensuring the voltage stability and power quality (PQ) of distribution systems regardless of the naturally intermittent characteristic of RE-based power generation. Wind power generation is currently one of the most promising RE sources because it can generate a considerable amount of electrical energy in a short period of time. Commonly used generators for wind turbine generators (WTGs) include permanent magnet synchronous generators (PMSGs) [1], doubly-fed induction generators (DFIGs) [2], and brushless wound-rotor doubly-fed generators [3]. Currently, most research papers on grid-connected PMSG-based WTGs (PMSG-WTGs) found in open literature have focused on advanced control schemes, low voltage ride-through (LVRT) capacity, and operating modes. M. Jamil et al. [4] suggested that, among commonly used generators in WTGs, PMSGs have more advantages compared with conventional DC and induction generators. The topologies of PMSG-WTG power converters were reviewed, including a thyristor-based inverter, hard-switched pulse with modulation (PWM) converter, multilevel converter, matrix converter, and Z-source inverter. For conventional small- and medium-size WTGs, power interfaces usually adopt the configuration that consists of a diode bridge rectifier and a buck boost converter. Although this configuration has the advantages of a simple circuit, low cost, and easy control, it causes a large amount of current harmonic and lowered power factor (PF) in the three-phase AC side, which leads to voltage pulses that lower PQ and efficiency, and shorten the life of the generator. A good WTG power interface must offer high conversion efficiency, low power consumption, PF correction, and low current harmonic. When a WTG is connected to a grid, appropriate control strategy is necessary to meet the power company’s LVRT specifications. On this basis, this study proposes a distributed control scheme to enhance LVRT capabilities of small- and medium-size PMSG-WTGs using silicon carbide (SiC)-based inverters.

Generally, PQ improvement is necessary for distribution systems embedded with DG systems because of the uncertainty and intermittency of RE sources, which can easily result in various undesirable effects, such as voltage sag. In [5], a grid-connected direct-drive PMSG-WTG model was simulated for various wind speeds and load conditions using PI-based controller. The results showed a properly regulated DC link voltage and output power. M. H. Qais et al. [6] reviewed LVRT capability enhancement methods for a grid-connected PMSG driven directly by a variable speed WT (VSWT). Later, the authors used a gray wolf optimizer (GWO) to tune eight PI controllers to improve LVRT capability and MPPT performance. In comparison with the genetic algorithm (GA) and simplex method, GWO algorithm yielded the best convergence, MPPT capability, and LVRT performance during symmetrical and asymmetrical faults [7]. In [8], a resistor-type superconducting fault current limiter (R-SFCL) was combined with superconducting magnetic energy storage (SMES) to improve the LVRT capability of a 2.5 MW PMSG-WTG. Improved transient stability and reduced cost for superconducting devices were achieved. P. Xing et al. [9] proposed a fast compositive control of LVRT for a 2 MW PMSG-WTG by consuming initial excessive energy with a crowbar circuit, and then converting excessive energy into rotor kinetic energy. In [10], a perturbation-observing nonlinear adaptive control was proposed for a dynamic voltage restorer (DVR) embedded with an energy storage system (ESS). This simple and robust control scheme does not require an accurate system model and full-state feedback. The results showed better performance than conventional fixed-gain vector control (VC) and feedback linearizing control based on an accurate system model. The authors used 10 MW PMSG- and DFIG-based WTGs to verify the control. M. Jahanpour-Dehkordi et al. [11] combined VC and direct torque control (DTC) using two hysteresis current controllers to achieve fast and smooth LVRT for a 2 MW PMSG-WTG. This method is better than DTC control and VC control in terms of smoothness and speed, respectively. In [12], the LVRT capability of a 2.5MVA full-scale PMSG-WTG was enhanced with active damping control and DC-link voltage bandwidth retuning. M. Nasiri and R. Mohammadi [13] enhanced the LVRT capability of a 1.5 MW PMSG-WTG with proposed back-to-back converter controllers and active power limiter without the need for external devices.

Some advanced control strategies for DFIG and PSMG wind turbines can be found in [14,15,16,17,18]. In [14], advanced control strategies were proposed to control the pitch angle, and the rotor and grid side converters of the doubly fed induction generator (DFIG)-based wind turbine (WT) for enhancing the LVRT capability. The converter systems used in the study include a back-to-back converter and a DC chopper. Simulation results showed that with the proposed control means, the LVRT capability of WT generators can be enhanced, and the oscillations in the stator and rotor currents can be effectively reduced. Both low-wind and high-wind speeds cases were investigated to verify the proposed control algorithm. With the same hardware system used in [14], the same group of authors of [15] proposed a LVRT control method to achieve the real-time regulation of the rotor’s excessive inertia energy. The reactive power capacity was simultaneously handled to satisfy the grid code. It was demonstrated with simulation results that the control topology or parameters can be changed at different stages of voltage fault period. In [16], a LVRT control scheme for the permanent-magnet synchronous generator (PMSG) with a back-to-back neutral-point-clamped, three-phase converter was proposed. In the studied grid voltage dip scenario, the proposed controllers designed for generator-side and grid-side converters can work concurrently to meet the LVRT requirement by storing the active power in the mechanical system of WT, and regulating the dc-link voltage at a constant value. Results from both simulation and experimental tests verified the effectiveness of the proposed method. The system stability issues of weak AC grid-connected DFIG-based wind turbines during LVRT were comprehensively investigated in [17]. To study the instability mechanism of a DFIG with back-to-back converter systems during a weak grid fault, a small signal state-space model was established. The effectiveness of the proposed LVRT control methodologies were validated with simulation and experiments. In [18], a wind speed combination model (WSCM) was proposed using the field-measured data, which was then used as equivalent wind speeds for each WTG when modeling the entire wind farm. Results from a case study verified the correctness of WSCM and the performance of the proposed algorithm in predicting the LVRT entering status of WT generators in the wind farm.

The use of a static synchronous compensator (STATCOM) for LVRT capability enhancement at the point of common coupling (PCC) was discussed in [19,20,21,22,23,24,25,26,27,28]. P. Dey et al. [19,20] developed a controller based on a pitch angle control and a flux weakening control and a STATCOM to enhance the LVRT capability of a PMSG-WTG. As a result, enhanced LVRT performance, reduced DC-link capacitor overvoltage, and the connection between the WTG and the grid during faults were achieved compared with conventional braking chopper-based LVRT strategy. In [21], a coordinated current control scheme was proposed for a 100 MW PMSG-WTG with a 20 MW STATCOM for severe LVRT by exchanging control roles between the two converters, such that the DC link voltage was controlled by the machine-side converter (MSC), and the grid active power was controlled by the grid-side converter (GSC). Synchronization was maintained, and reactive power requirement was satisfied. J. Yao et al. [22] studied a cost-effective capacity configuration strategy for the improvement of LVRT capability of a hybrid wind farm consisting of a 30 MW fixed-speed induction generator (FSIG) in conjunction with a 30 MW PMSG by using the PMSG as a STATCOM during grid fault. For LVRT capability improvement of DFIG-WTGs using STATCOMs, a PI controller with robust control technique was proposed in [23], where the reliability of the WTG was ultimately increased, and a fuzzy adaptive proportional-integral-derivative (PID) controller was proposed in [24], with enhanced control accuracy and performance. For self-excited induction generator (SEIG)-WTGs, M. I. Mosaad et al. [25] proposed a model reference adaptive control (MRAC) of STATCOM, yielding more efficient and more robust performance than that of a GA-tuned PI controller. In [26], the LVRT capability enhancement of STATCOM for the terminal voltage of a squirrel cage induction generator (SCIG)-WTG was studied in a wind farm in Bizerte, Tunisia. T. Tanaka et al. [27,28] used an 80MVar/33kV STATCOM based on multilevel converters for LVRT capability enhancement of non-specific-type offshore WTGs.

Most of the above reviewed applications of STATOM for LVRT capability enhancement adopted the configuration of centralized compensation, where a single STATCOM module was employed at the PCC to take charge of the control goal. However, this configuration is expensive, and the required system capacity is normally high. Moreover, if a single malfunction occurs in the STATCOM, then the LVRT capability of the system may deteriorate drastically. In this regard, this study aims to propose a distributed LVRT compensator (LVRTC) for every separate WTG. The use of distributed LVRTCs increases reliability and flexibility, while reducing the required capacity and overall costs. In the next section, LVRT specifications will be explained followed by the proposed distributed LVRTC and WTG system configuration. The mathematical models required for the controller design using a dq-axis current decoupling algorithm and quantitative design of the three-phase inverter controller are explained in Section 3. In Section 4, typical results of simulation in a computer software environment and hardware tests using TI’s DSP as control core are presented. Finally, the conclusion of this work is summarized in the last section.

## 2. LVRT Specifications and WTG System Configuration

### 2.1. LVRT Specifications

In a wind farm, the stability and reliability of the system are two of the most important factors for operation. When disturbances occur, it is required that a grid-connected WTG doesn’t simply disconnect from the grid, such that the power system has a chance to maintain its voltage stability. As a result, a WTG must be able to feed appropriate active power and reactive power to restore the system voltage and frequency back to nominal values. In other words, modern grid-connected WTGs must be equipped with reactive power controls and the ability to eliminate faults, especially for cases where the penetration of wind power generation is high. Specifications of grid-connected WTGs include frequency range, voltage tolerance, and LVRT capability. Among these specifications, LVRT capability is the most important criterion in a WTG embedded system.

**Figure 1:**
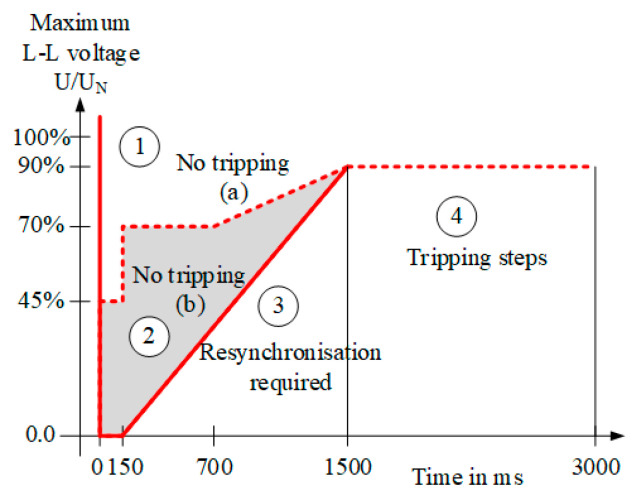
Requirements of low-voltage tolerance for WTGs by E.ON. (U denotes the system voltage, U_N_ denotes the system nominal voltage).

**Figure 2:**
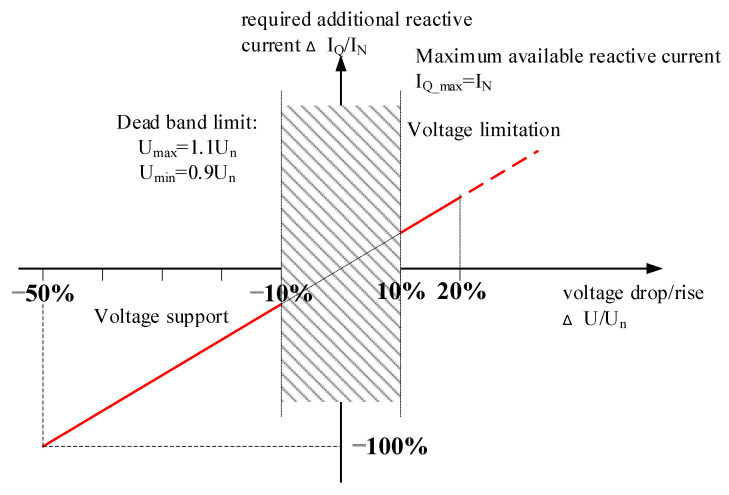
Requirements of reactive current compensation for WTGs by E.ON. (U denotes the system voltage, U_N_ denotes the nominal voltage of the system; I_Q_ denotes the reactive current; I_N_ denotes the nominal current of the LVRTC in this study).

To regulate and facilitate the development of various RE generations, German electric utility company, E.ON, has set tolerance requirements for newly installed WTGs, as shown in Figure 1 [29]. In Figure 1, when the grid voltage drops to zone 1 or zone 2, the WTG has to maintain the connection with the grid. For voltage restoration, if the grid voltage drops to zone 2, and the fault is later removed, the WTG must restore the power with a rate of 10% per second. In zone 3, the WTG is allowed to be disconnected from the grid to protect the generator, and when the fault is removed, the WTG must be reconnected with the grid, and restore the power with a rate of 20% per second. If the failure continues for more than 1.5 s, the WTG can be protected from the network through disconnection. Moreover, the WTG should also provide reactive power, as conventional power systems do, to help stabilize and restore the voltage, as shown in Figure 2 [29]. As can be seen in Figure 2, when the voltage drops out of the non-action zone (±10%), the required percentage of reactive current from the WTG is two times the percentage of the voltage sag. For example, when the voltage drop is 50%, the WTG must provide reactive current of 100% system capacity within 20ms, as shown in Equation (1). In Equation (1), $i_{fq}^{*}$ denotes the per unit value of the reactive current command in q-axes, $V_{dN}$ is the d-axes system nominal voltage, and $V_{d}$ represents the d-axes monitored system voltage.

(1) $$i_{fq}^{*}\left(pu\right)=2\left(\frac{V_{dN}-V_{d}}{V_{dN}}\right)\left(pu\right)$$

### 2.2. WTG Interface

**Figure 3:**
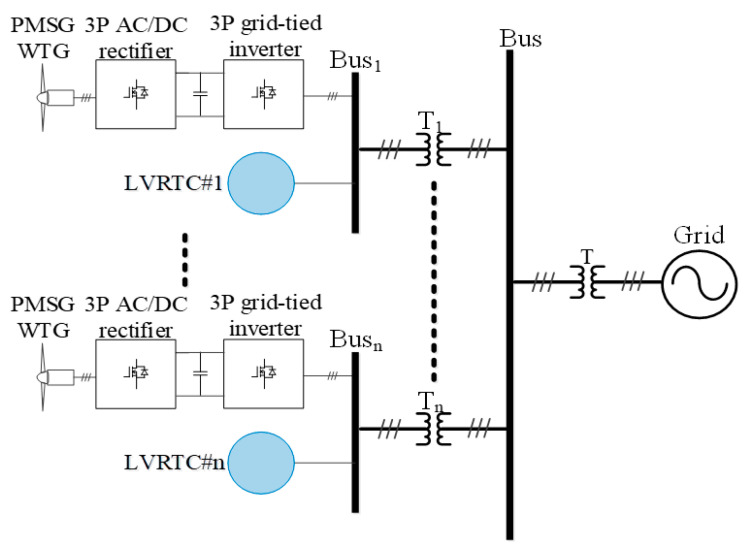
Conceptual WTG schematic architecture and allocation of the proposed LVRTC.

Figure 3 shows schematic system architecture of the proposed distributed LVRTC system investigated in this study.

Mathematical Model Derivation of Grid-connected Three-phase Inverter. 

**Figure 4:**
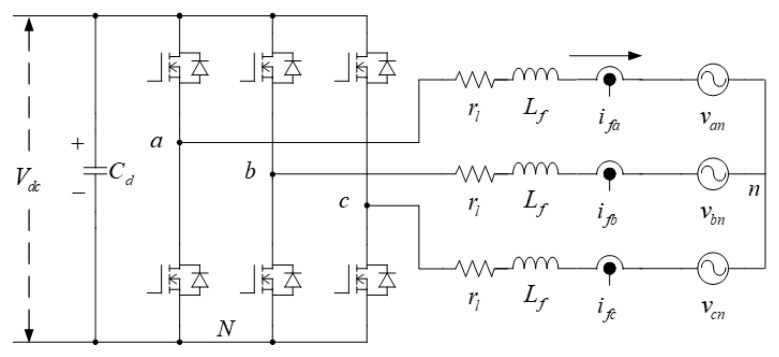
Schematic architecture of the proposed LVRTC on a three-phase grid-connected inverter.

The control goal of the proposed LVRTC using a three-phase grid-connected inverter system shown in Figure 4 is to maintain the DC link voltage, and output the reactive power as commanded. In Figure 4, $L_{f}$ is the filter inductance; $i_{fa}$, $i_{fb}$, $i_{fc}$ are, respectively, the three currents of the output filter; $r_{l}$ denotes the equivalent resistance of the inductor; $v_{an}$, $v_{bn}$, $v_{cn}$ are, respectively, the three phase-voltages of the system; $C_{d}$ is the dc capacitor of the inverter. The inverter model for the related controller design can be established according to Kirchhoff’s laws. The three-phase coordinate system (here assumed to be positive phase sequence) is converted into a synchronous rotating coordinate system.

In this study, pulse width modulation (PWM) is adopted for power switching, where three-phase sine waves are used. The switching equation can be expressed as follows:

(2) $${e_{iN}=\left(\frac{1}{2}+k_{pwm}\right)V_{dc}|}_{i=a,b,c},$$

where *k_pwm_* is constant and defined as *V_dc_/*2*V_tri_*, and *V_tri_* represents the triangular carrier wave. Assuming that the three-phase power supply is balanced, dq-axis voltages are as follows: *v_d_* = *V_m_*, and *v_q_* = 0, where *V_m_* represents the grid peak phase voltage. As a result, active and reactive powers are as follows: *P_ac_* = 3*V_m_i_fd_*/2, and *Q_ac_* = 3*V_m_i_fq_*/2.

## 3. Controller Design for LVRTC

**Figure 5:**
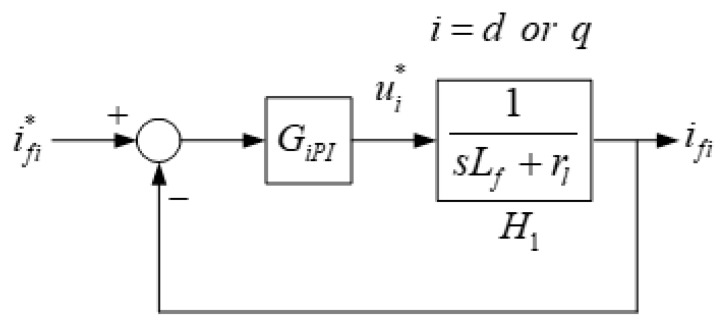
Schematic architecture of the current controller.

To achieve a better dynamic performance, the proposed LVRTC on a three-phase inverter adopts a dual-loop control scheme, where the inner loop controls the output currents, and the outer loop controls the DC voltage and reactive power. When the WTG outputs power, the DC voltage controller regulates the DC link voltage to follow the voltage command, such that the link stays unaffected by the control of reactive power. Under normal circumstances, higher power factor and low-harmonic currents are required to reduce losses, and improve system stability. With a proper mathematical derivation, system voltage and current equations can be decoupled into d and q axes, as shown as follows:

(3) $$u_{d}=-v_{d}+\omega_{e}L_{f}i_{fq}+e_{d};$$

(4) $$u_{q}=-v_{q}+\omega_{e}L_{f}i_{fd}+e_{q},$$

where *u_d,q_* represents the inverter switching node dq-axis voltage command, *v_d,q_* represents grid dq-axis voltage, *L_f_* is the filter inductance, $r_{l}$ denotes the equivalent resistance of the inductor. *I_fd,q_* represents dq-axis filter current, and *e_d,q_* represents inverter switching node dq-axis voltages. After derivation, we obtain the plant transfer function and dq-axis current controller transfer function as follows:

(5) $$H_{1}=\frac{1}{sL_{f}+r_{1}};$$

(6) $${G_{iPI}=\frac{k_{iP}s+k_{iI}}{s}=k_{iP}\left(1+\frac{1}{T_{i}s}\right)|}_{i=d,q},$$

where *k_iP_* represents the proportional gain, *k_iI_* represents the integral gain, and *T_i_* represents the integral constant and is defined as *k_iP_*/*k_iI_*. The inner loop current control structure is shown in Figure 5.

As shown in Figure 5, the close-loop transfer function of the current controller can be derived as follows:

(7) $${\frac{i_{fi}}{i_{fi}^{*}}=\frac{\frac{k_{iP}\left(T_{i}s+1\right)}{T_{i}s}\frac{1}{r_{l}\left(T_{j}s+1\right)}}{1+\frac{k_{iP}\left(T_{i}s+1\right)}{T_{i}s}\frac{1}{r_{l}\left(T_{j}s+1\right)}}|}_{i=d,q},$$

where *T_j_* represents the time constant, and is defined as *L_f_*/*r_l_*. Letting *T_d_* = *T_q_* = *T_j_*, we obtain the following:

(8) $$\frac{i_{fd}}{i_{fd}^{*}}=\frac{1}{\left(L_{f}/k_{dp}\right)s+1};$$

(9) $$\frac{i_{fq}}{i_{fq}^{*}}=\frac{1}{\left(L_{f}/k_{qp}\right)s+1}.$$

**Figure 6:**
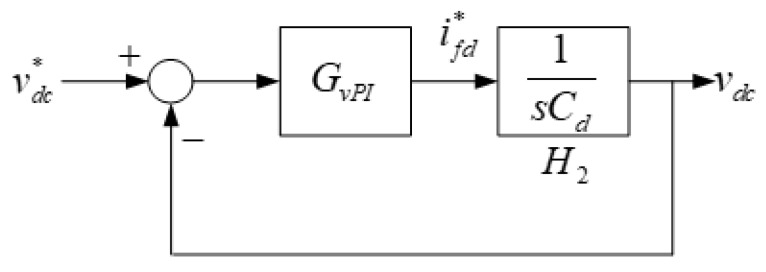
Schematic architecture of the DC voltage controller.

The DC voltage controller is constructed as shown in Figure 6, where the DC voltage error is used to obtain the current command through controller *G_vPI_*. Controller and plant transfer functions are expressed as follows:

(10) $$H_{2}=\frac{1}{sC_{d}};$$

(11) $$G_{vPI}=\frac{k_{vP}s+k_{vI}}{s}=k_{vP}\left(1+\frac{1}{T_{v}s}\right),$$

where $C_{d}$ is the dc capacitor of the inverter. *k_vP_* represents proportional gain, *k_vI_* represents integral gain, and *T_v_* represents integral constant, and is defined as *k_vP_*/*k_vI_*. The control structure is shown in Figure 6.

**Figure 7:**
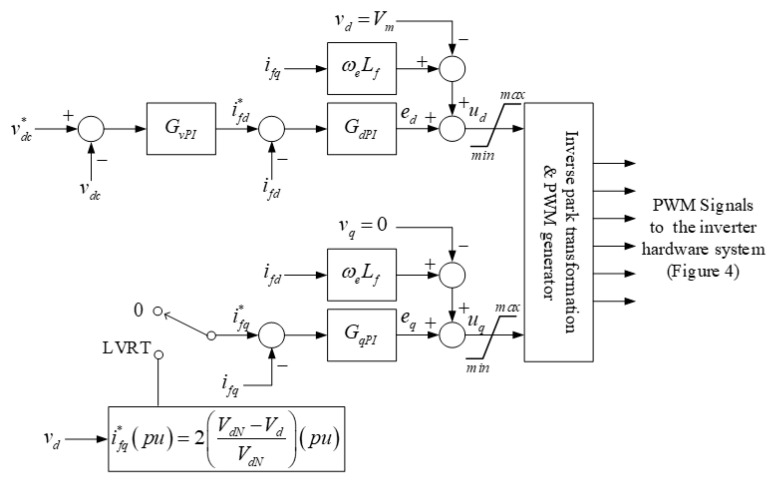
Schematic architecture of the complete LVRTC controller ($\omega_{e}$ denotes the angular frequency of the system).

Based on Figure 6, the close-loop transfer function of the DC voltage controller can be expressed as shown in Equation (12). As a result, the complete inverter controller can be obtained as shown in Figure 7.

(12) $$\frac{v_{dc}}{v_{dc}^{*}}=\frac{\frac{k_{vP}\left(T_{v}s+1\right)}{T_{v}s}\frac{1}{C_{d}s}}{1+\frac{k_{vP}\left(T_{v}s+1\right)}{T_{v}s}\frac{1}{C_{d}s}}.$$

As shown in Figure 2 and Figure 7, when the grid voltage falls below 90% of the nominal value, the reactive current control command will be switched to LVRT mode, and perform reactive power command tracking control. In this study case, the system specifications of the inverter are as follows: system rating: 2 kVA; grid voltage *V_L-L_*: 220 V/60 Hz; DC voltage *V_dc_*: 400 V; filter inductance *L_f_*: 3 mH; filter inductor resistance *r_l_*: 3.5 Ω; DC link regulator capacitance *C_d_*: 560 μF; and switching frequency *f_SW_*: 18 kHz. According to these specifications, the plant transfer functions of the DC voltage loop and current loop are as follows:

(13) $$H_{1}=\frac{1}{0.003s+3.5};$$

(14) $$H_{2}=\frac{1}{5.6\times 10^{-4}s}.$$

**Figure 8:**
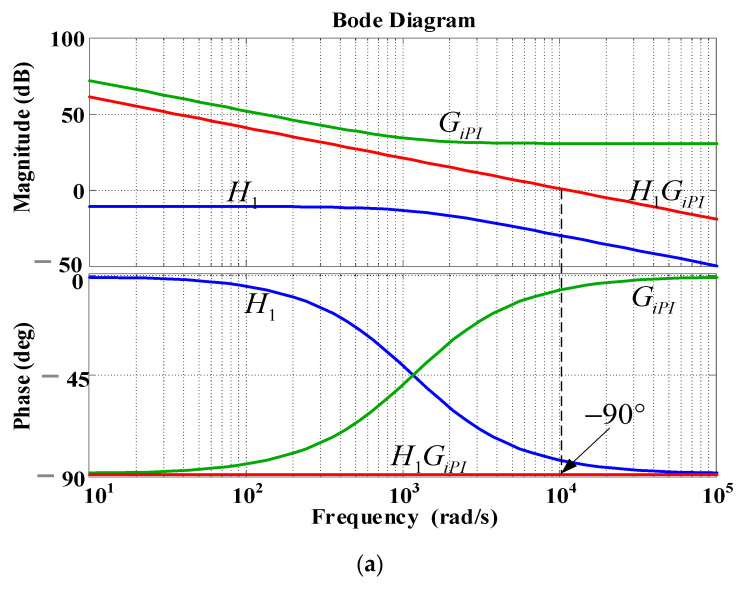

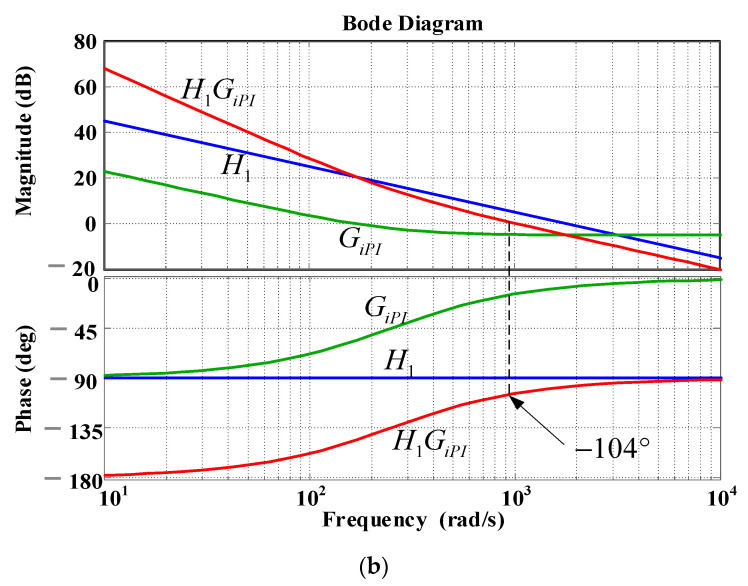
Bode plots of designed controller: (**a**) inner loop current controller; (**b**) DC voltage controller.

In this application case, a dual-loop control scheme is used to achieve a better voltage regulation on the DC bus. Theoretically, the outer loop (the DC voltage controller) should have a smaller bandwidth than the inner loop (the current controller). In practice, these parameters are properly chosen to achieve the desired dynamic performance [30]. Here, the close-loop bandwidth of the DC voltage controller is set at 500 Hz, and the bandwidth of the current response is set at 1/10 times the switching frequency, which is 1.8 kHz. This approach yields proportional and integral gains of the current control loop: *k_dP_* = *k_qP_* = 33.93, and *k_dI_* = *k_qI_* = 39584. Next, Equation (12) can be expressed in the form of a second order standard equation, with damping ratio *ξ*, and undamped natural frequency *ω_0_*, as shown as follows:

(15) $$s^{2}+2\xi \omega_{0}s+\omega_{0}^{2}=0,$$

where *k_vP_* = 2*ξω*_0_*C_d_*, *T_v_* = 2*ξ*/*ω*_0_, and, *k*_v*P*_ = *k_vP_*/*T_v_*. *ξ* and *ω*_0_ can be chosen according to the desired dynamic performance. Letting *ξ* = 1 and *ω*_0_ = 500 yields the following results: *k_vP_* = 0.56, and *k_vI_* = 140. Figure 8 shows the Bode plots of the DC voltage loops.

## 4. Simulation and Implementation

**Figure 9:**
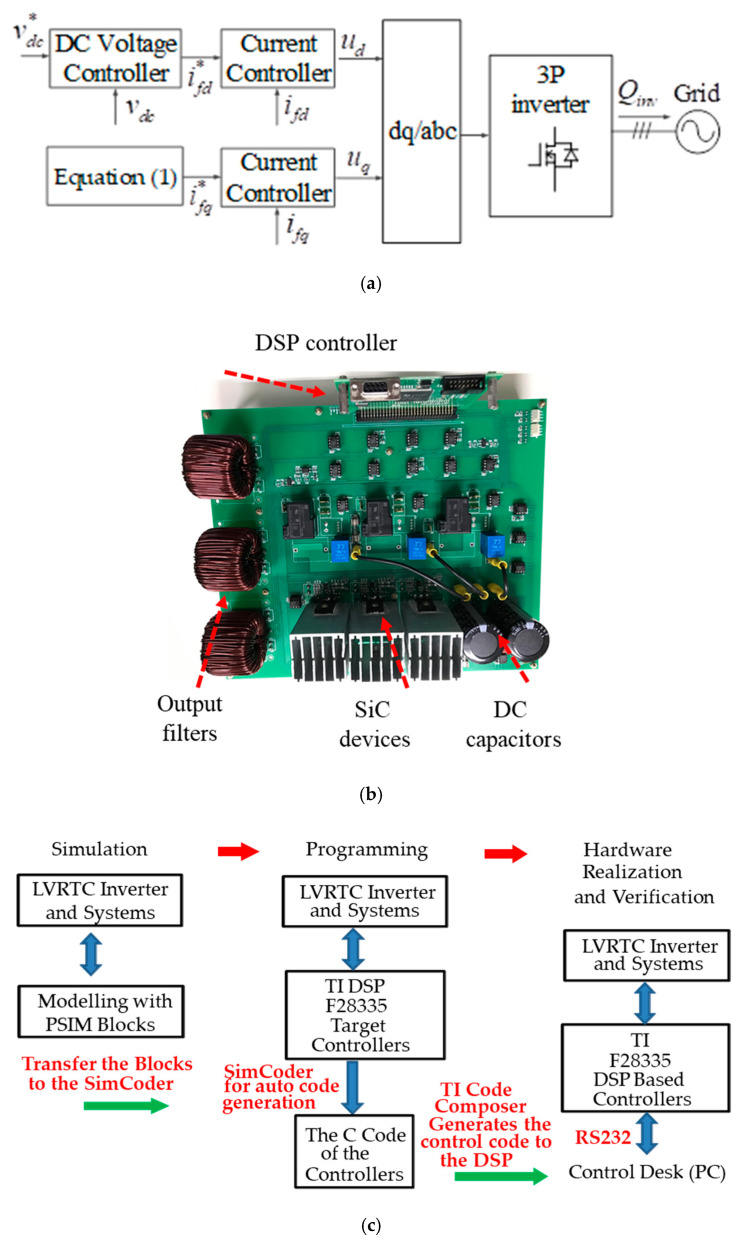
(**a**) Schematic of simulated grid-connected LVRTC; (**b**) the photograph of the LVRTC SiC-based 3P inverter circuit prototype; (**c**) the DSP programming steps in hardware implementation of LVRTC.

**Figure 10:**
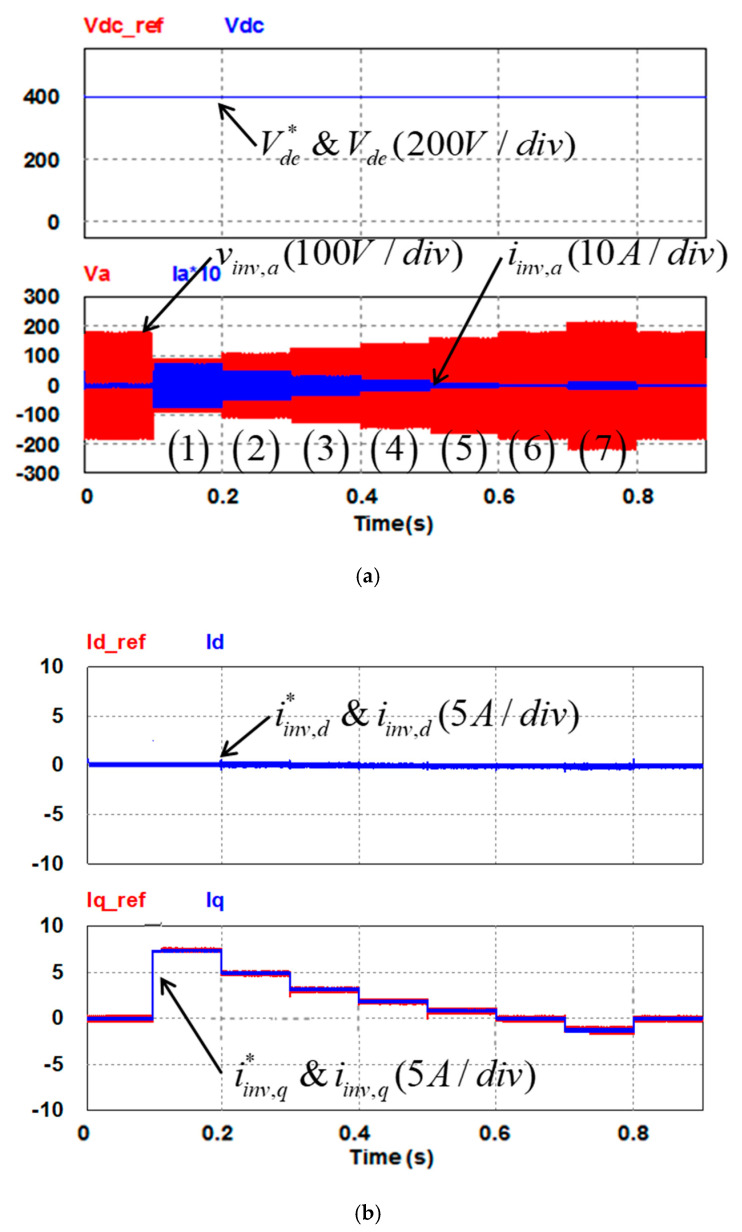
Simulation results of LVRT capability: (**a**) DC voltage and its command signal/grid phase A voltage and current (20 times); (**b**) inverter d-axis current and its command/q-axis current and its command.

**Figure 11:**
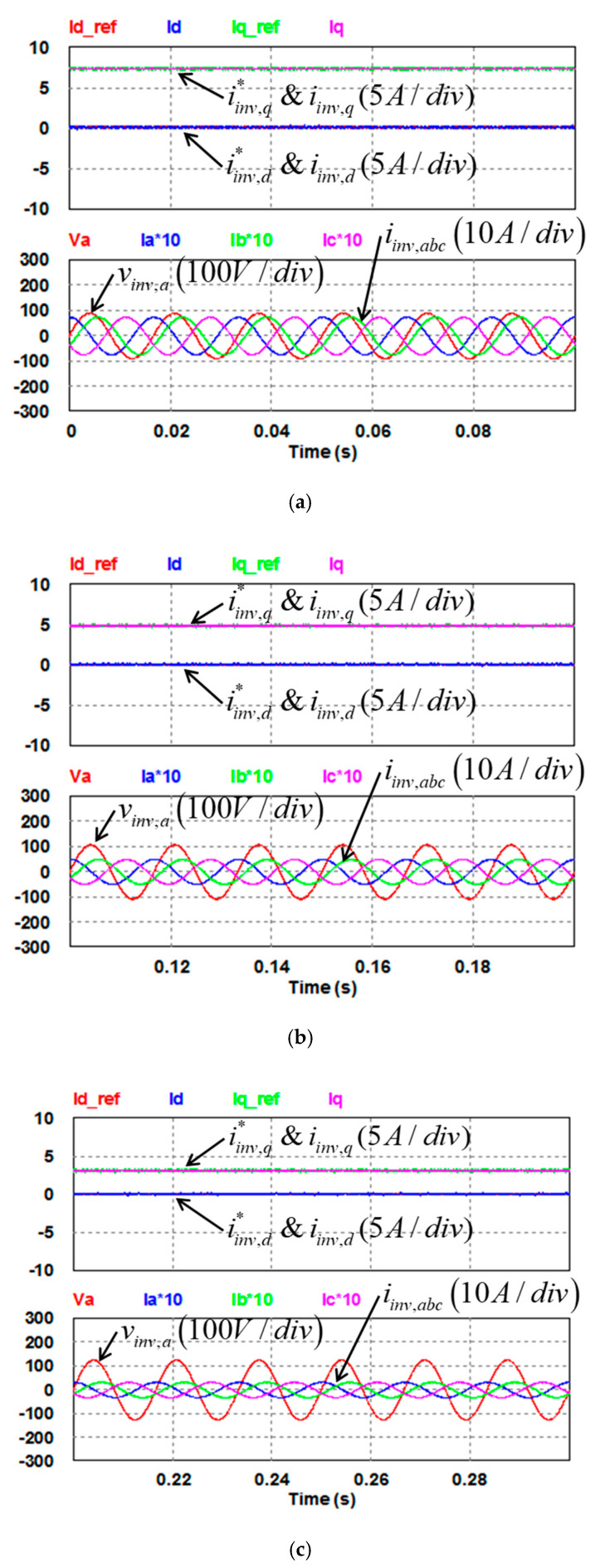

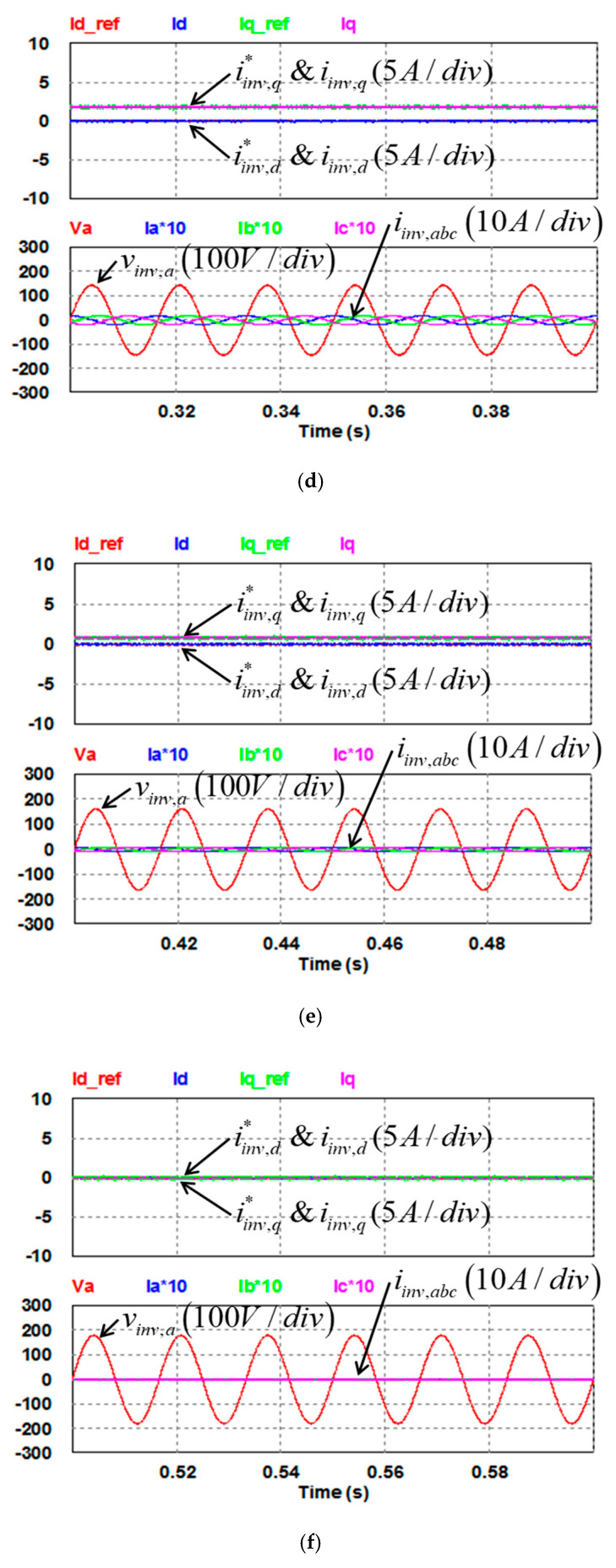

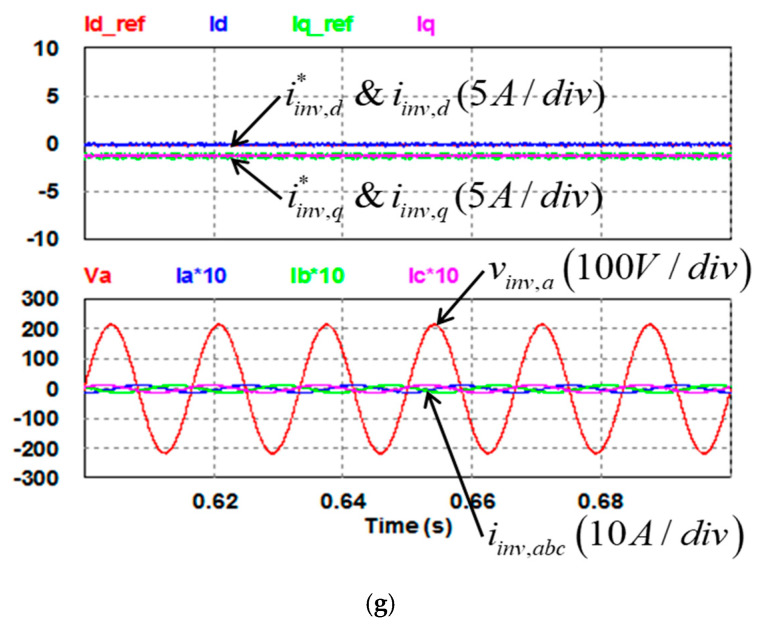
Detailed view of inverter dq-axis currents and their commands/phase A voltage (V) and three-phase currents (A) in Figure 10: (**a**) stage (1); (**b**) stage (2); (**c**) stage (3); (**d**) stage (4); (**e**) stage (5); (**f**) stage (6); (**g**) stage (7).

**Figure 12:**
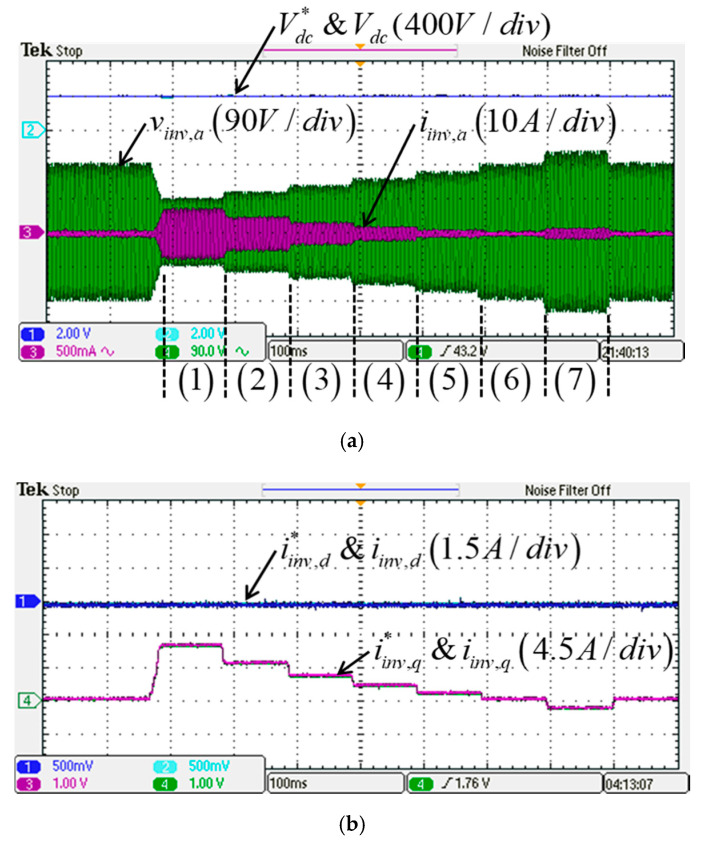
Implementation results of LVRT capability: (**a**) DC voltage and its command signal (400 V/div)/grid phase A voltage (90 V/div) and current (5 A/div); (**b**) inverter d-axis current and its command (1.5 A/div)/q-axis current and its command (4.5 A/div).

**Figure 13:**
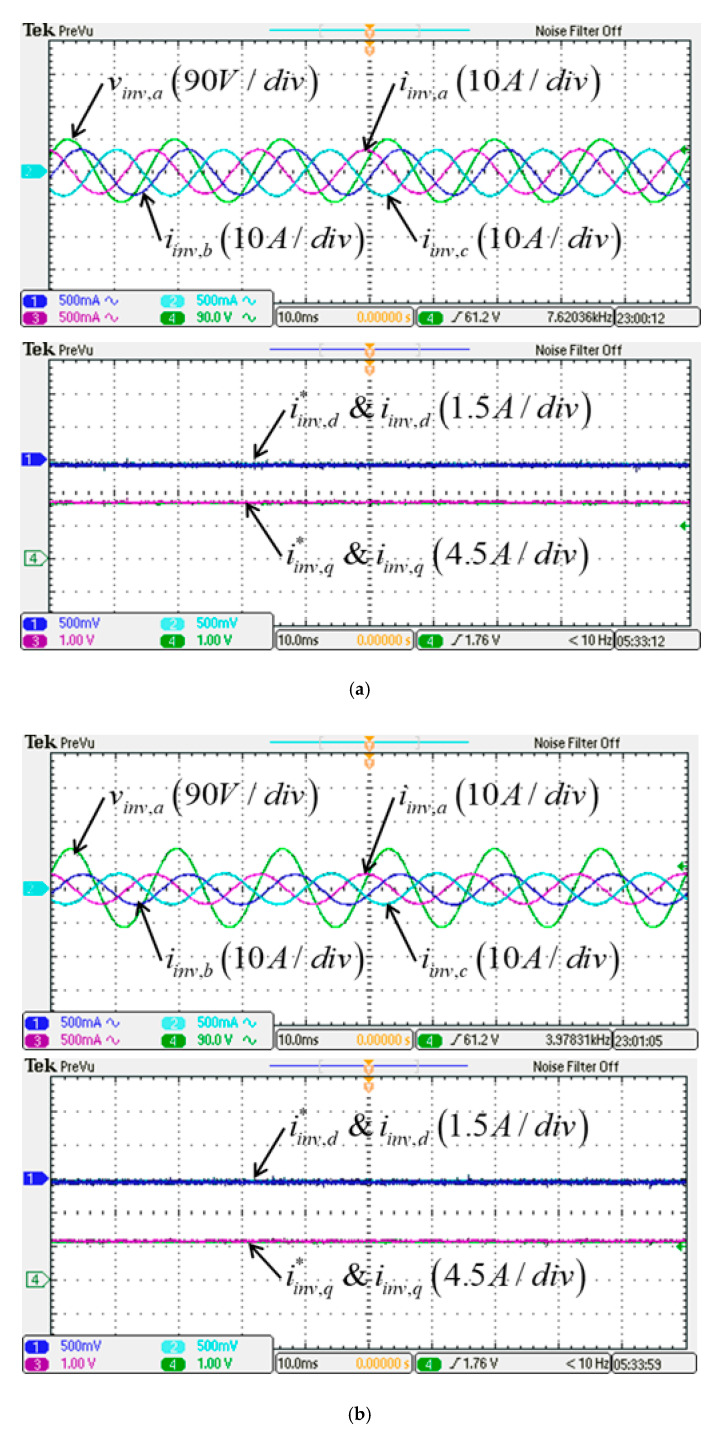

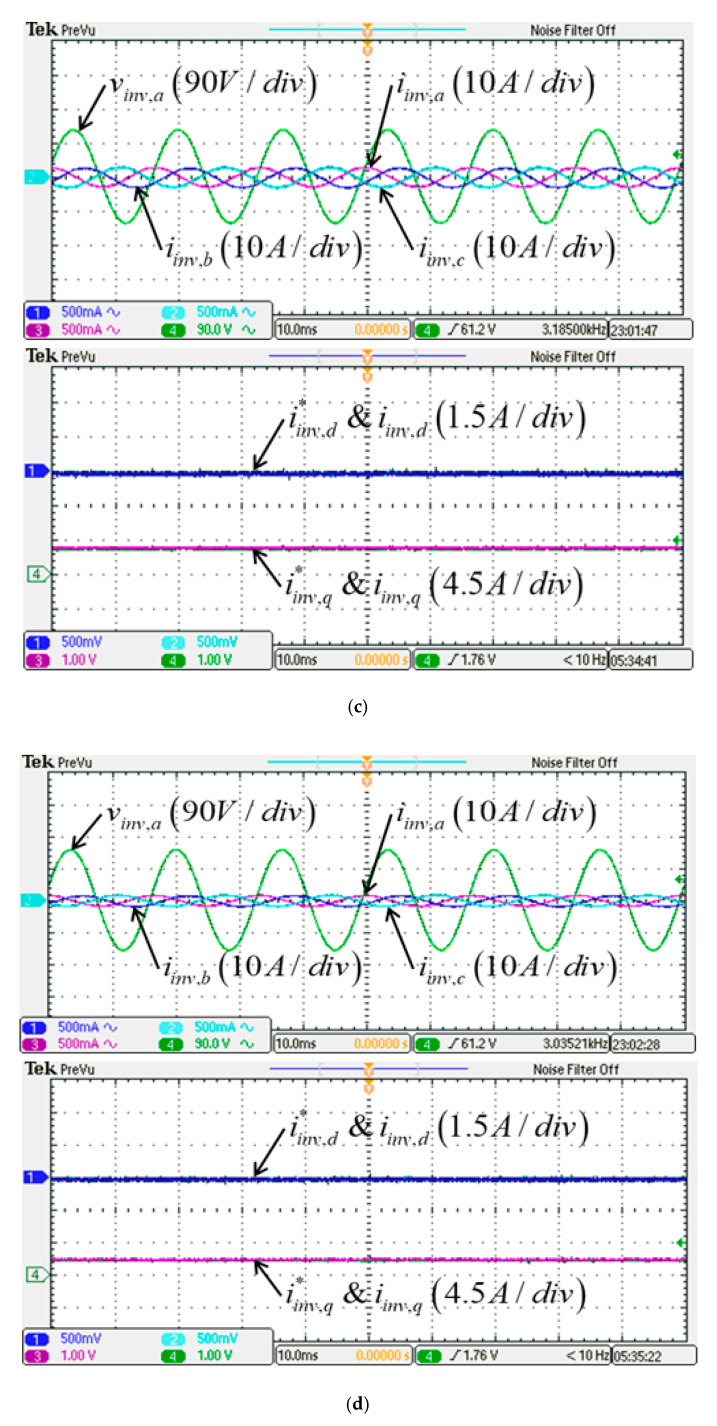

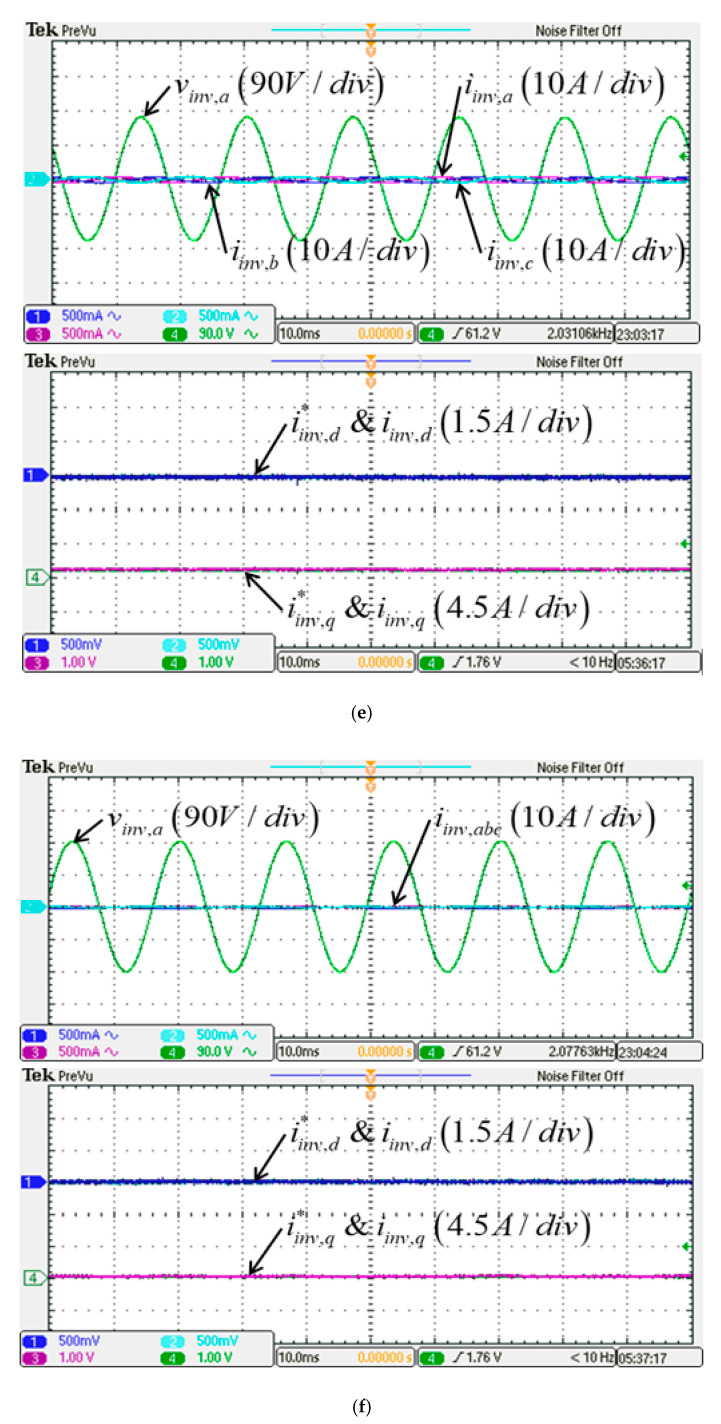

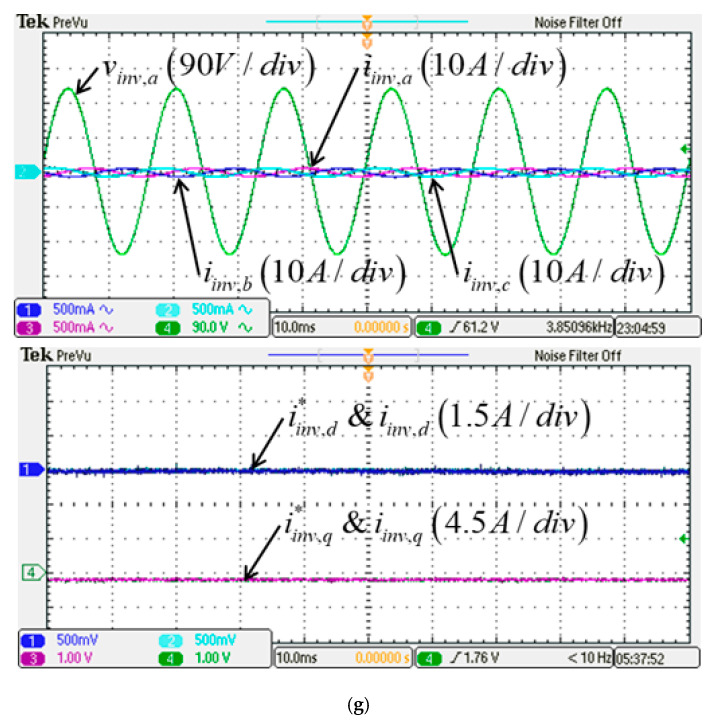
Detailed view of phase A voltage and three-phase currents/inverter dq-axis currents and their commands in Figure 12: (**a**) stage (1); (**b**) stage (2); (**c**) stage (3); (**d**) stage (4); (**e**) stage (5); (**f**) stage (6); (**g**) stage (7).

In this study, the POWERSIM simulation software is used in both simulation and hardware implementation, and a personal computer equipped with an i7-9700 CPU, @ 3.00 GHz is used as a control desk. The sampling frequency used in the hardware design of the proposed LVRTC is 50 kHz. It is the same as that of the switching frequency used. Figure 9a shows the schematic architecture of the simulated LVRTC system connected to the power grid. For the simulation scenario, the grid voltage variation sequence is planned as follows: 0.5 pu, 0.6 pu, 0.7 pu, 0.8 pu, 0.9 pu, 1.0 pu, 1.2 pu, and then 1.0 pu. The corresponding reactive power commands for the LVRTC are generated according to the grid code. The simulation results are shown in Figure 10. Figure 11 shows the detailed steady-state waveforms of Figure 10. It can be observed that the LVRTC successfully feeds appropriate reactive current to the grid during faults. Figure 12 shows the results of the experimental tests using the constructed 2 kVA SiC-based three-phase inverter as shown in Figure 9b, and a programmable AC power supply emulating the grid. In this study, the TI’s DSP (TMS320F28335) is used as the control core for the proposed LVRTC hardware, in which six SiC switching devices using ON Semiconductor’s NTHL060N090SC1 with the driver integrated circuits of Si8271 and the sensor devices for sensing currents and voltages are used to facilitate a flexible experimental system. Regarding the task of DSP’s programming, the SimCoder tool of POWERSIM software (2021) is used to convert the AD modules, various functional blocks, and controllers into the required DSP control program. Then, the DSP control program can be burned into the DSP chip. In the hardware implementation stage, the control desk (PC) is used to communicate with the DSP, and the controller parameters can be adjusted online to achieve the best control performance and accuracy. Figure 9c shows the DSP programming steps in hardware implementation of LVRTC. In this study, the identical system condition used in the simulation case is implemented here in the hardware test. Figure 13 shows the detailed steady state waveforms of Figure 12. It can be clearly observed that the implementation results are in good agreement with the simulation results.

## 5. Discussion

With the growth of wind power generation over the past decade, various LVRT technologies have been proposed to ensure the safe and reliable operation of WTGs and power systems. Some advanced LVRT control algorithms reviewed in this paper and reported in many other similar papers in the literature can be categorized into two groups, i.e., the protection strategies- and the control strategies-based methodologies. However, most of the reported control strategies were implemented by the WTG built-in converters with complex controllers. It should be noted that with a limited hardware capacity, during the period of fault, the real power output capability of the built-in converters of WTGs must be dynamically limited to meet real-time reactive power requirements, and comply with strict grid codes. Based on the LVRT grid code, when the voltage dipped over 0.5 pu, the total current capacity of inverter system of the WTGs should be used to output the reactive power. This can strongly limit the control flexibility of a real power restoration scheme in the post-fault period. In this paper, we have demonstrated a distributed LVRT compensator (LVRTC) control scheme that can, to some degree, solve the above problem, and increase the system control flexibility. In addition, with the proposed distributed LVRTC control scheme, the WTG in a wind farm has greater freedom in performing its protection schemes, or regulating its power flow to ensure system stability.

## 6. Conclusions

This study has proposed a new control scheme regarding distributed LVRTC and real-time reactive power compensation functions for the operation of WTGs in a wind farm according to E.ON’s specifications. The proposed compensation scheme using distributed LVRTCs can effectively enhance reliability, as well as reduce cost compared with centralized LVRT compensators. In addition, with the help of the proposed LVRTC scheme, WTGs can continuously output real power within their maximum current limit during the period of voltage drop, which is a great merit for the secure operation of modern power distribution systems embedded with WTGs. In this study, a complete design example of LVRTCs using silicon carbide (SiC), a wide band gap (WBG) semiconductor, based three-phase voltage source inverter has been demonstrated. The mathematical models of inverter systems have been derived using the dq-axis decoupling method to enable separate control of active and reactive powers. On the basis of the results obtained from the simulation and hardware tests, when a voltage fault occurs, the proposed control scheme can achieve a good regulation of LVRTCs’ DC bus voltage, and the real-time compensation of appropriate reactive current according to the grid code to help restore the nominal grid voltage.

## Data Availability

No new data were created or analyzed in this study. Data sharing is not applicable to this article.
